# Crystal structure of 4,6-di­chloro-5-methyl­pyrimidine

**DOI:** 10.1107/S2056989015024020

**Published:** 2015-12-19

**Authors:** Meriem Medjani, Noudjoud Hamdouni, Ouarda Brihi, Ali Boudjada, Jean Meinnel

**Affiliations:** a Laboratory of Crystallography, Department of Physics, University Mentouri Brothers Constantine, 25000 Constantine, Algeria; b UMR 6226 CNRS University of Rennes 1 ‘Chemical Sciences Rennes’, ‘Team Systems and Synthetic Condensed Electroactive’, 263 Avenue du General Leclerc, F-35042 Rennes, France

**Keywords:** crystal structure, pyrimidine, inversion dimers, C—H⋯N hydrogen bonding

## Abstract

The title compound, C_5_H_4_Cl_2_N_2_, is essentially planar with an r.m.s. deviation for all non-H atoms of 0.009 Å. The largest deviation from the mean plane is 0.016 (4) Å for an N atom. In the crystal, mol­ecules are linked by pairs of C—H⋯N hydrogen bonds, forming inversion dimers, enclosing an *R* ^2^_2_(6) ring motif.

## Related literature   

For the applications of pyrimidine derivatives as pesticides and pharmaceutical agents, see: Condon *et al.* (1993 ▸); as agrochemicals, see: Maeno *et al.* (1990 ▸); as anti­viral agents, see: Gilchrist (1997 ▸); as herbicides, see: Selby *et al.* (2002 ▸); Zhu *et al.* (2007 ▸); and for applications of organoselenide compounds, see: Ip *et al.* (1997 ▸). For the crystal structure of 5-methyl­pyrimidine, see: Furberg *et al.* (1979 ▸).
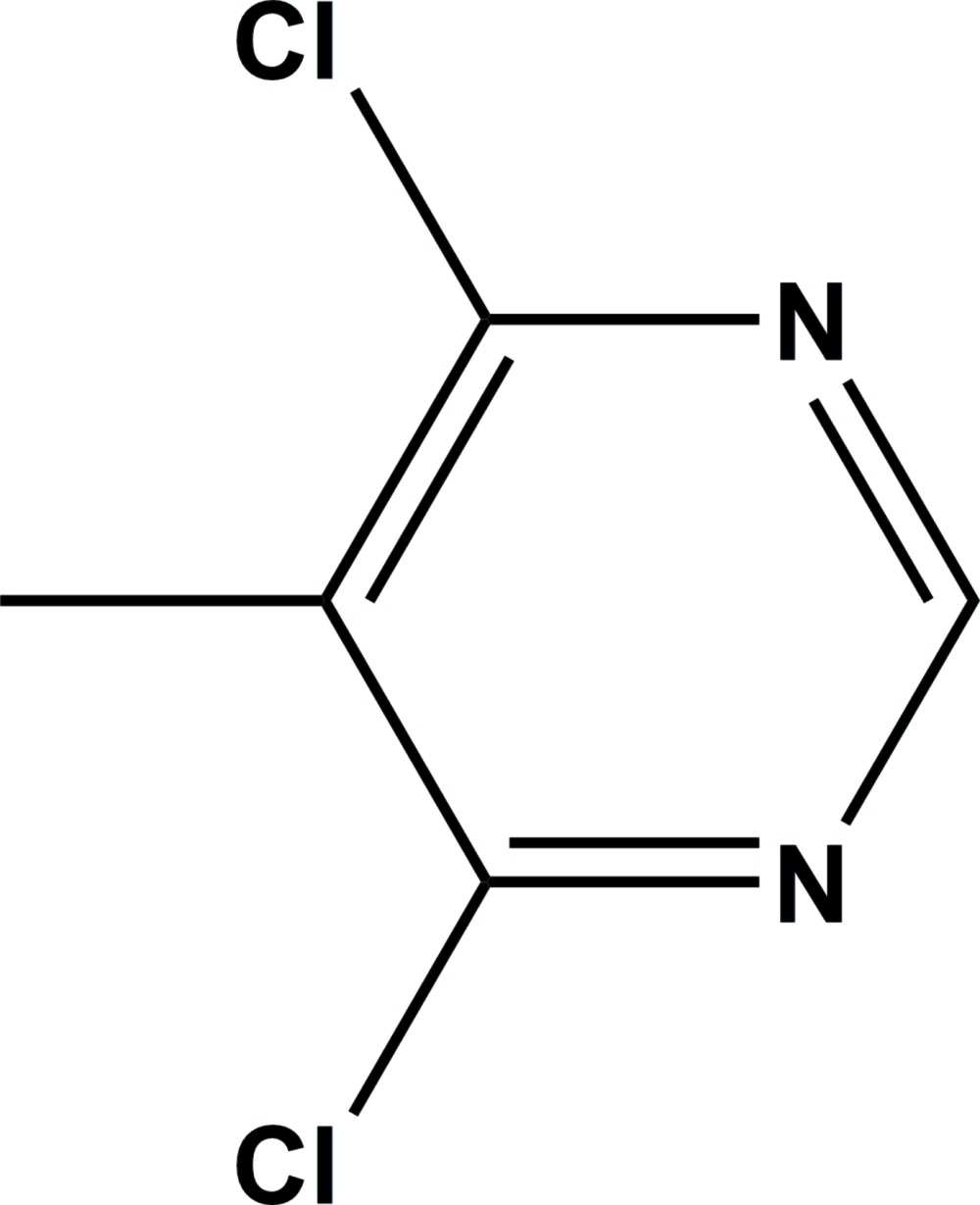


## Experimental   

### Crystal data  

C_5_H_4_Cl_2_N_2_
*M* *_r_* = 163.00
Monoclinic, 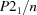

*a* = 7.463 (5) Å
*b* = 7.827 (5) Å
*c* = 11.790 (5) Å
β = 93.233 (5)°
*V* = 687.6 (7) Å^3^
*Z* = 4
Mo *K*α radiation
μ = 0.85 mm^−1^
*T* = 293 K
0.11 × 0.10 × 0.08 mm

### Data collection  

Oxford Diffraction Xcalibur, Eos diffractometer
Absorption correction: multi-scan (*CrysAlis PRO*; Oxford Diffraction, 2013 ▸) *T*_min_ = 0.922, *T*_max_ = 0.934
2347 measured reflections
1228 independent reflections
791 reflections with *I* > 2σ(*I*)
*R*_int_ = 0.099

### Refinement  

*R*[*F* ^2^ > 2σ(*F* ^2^)] = 0.068
*wR*(*F* ^2^) = 0.173
*S* = 1.01
1228 reflections
83 parameters
H-atom parameters constrained
Δρ_max_ = 0.39 e Å^−3^
Δρ_min_ = −0.38 e Å^−3^

### 

Data collection: *CrysAlis PRO* (Oxford Diffraction, 2013 ▸); cell refinement: *CrysAlis PRO*; data reduction: *CrysAlis PRO*; program(s) used to solve structure: *SIR97* (Altomare *et al.*, 1999 ▸); program(s) used to refine structure: *SHELXL2014*/6 (Sheldrick, 2015 ▸); molecular graphics: *PLATON* (Spek, 2009 ▸) and *Mercury* (Macrae *et al.*, 2008 ▸); software used to prepare material for publication: *SHELXL2014*/6 and *PLATON*.

## Supplementary Material

Crystal structure: contains datablock(s) global, I. DOI: 10.1107/S2056989015024020/su5261sup1.cif

Structure factors: contains datablock(s) I. DOI: 10.1107/S2056989015024020/su5261Isup2.hkl

**Fig. 1.**
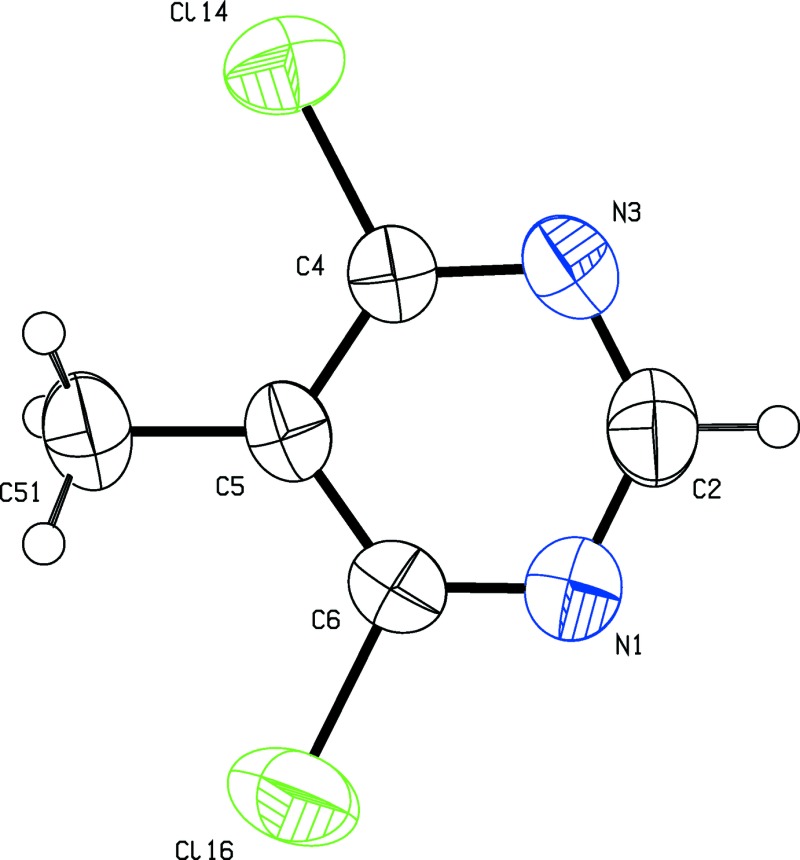
The molecular structure of the title compound, with atom labelling. Displacement ellipsoids drawn at the 50% probability level.

**Fig. 2.**
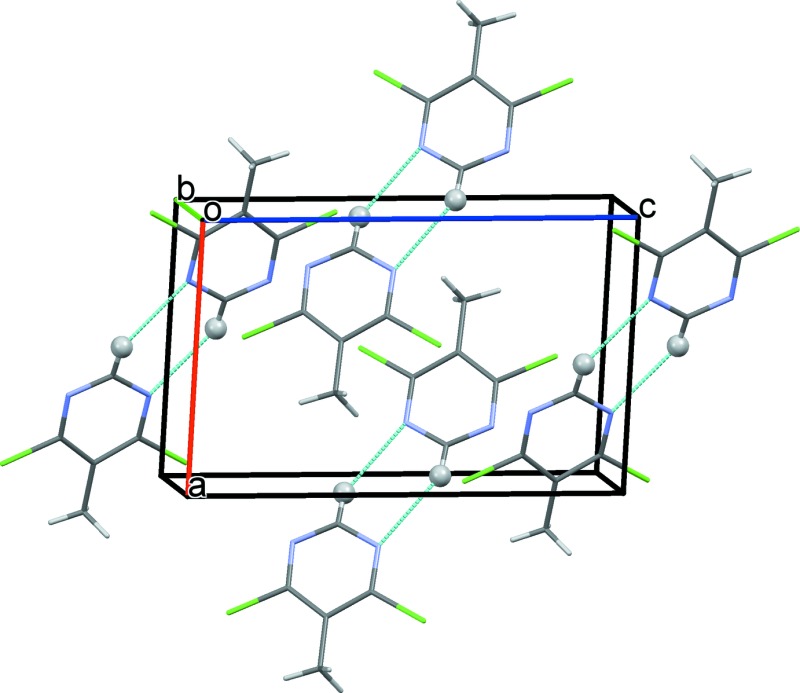
The crystal packing of the title compound, viewed along the b axis. Hydrogen bonds are shown as dashed lines (see Table 1).

. DOI: 10.1107/S2056989015024020/su5261fig1.tif
The mol­ecular structure of the title compound, with atom labelling. Displacement ellipsoids drawn at the 50% probability level.

b . DOI: 10.1107/S2056989015024020/su5261fig2.tif
The crystal packing of the title compound, viewed along the *b* axis. Hydrogen bonds are shown as dashed lines (see Table 1).

CCDC reference: 1442378

Additional supporting information:  crystallographic information; 3D view; checkCIF report

## Figures and Tables

**Table 1:** Hydrogen-bond geometry (Å, °)

*D*—H⋯*A*	*D*—H	H⋯*A*	*D*⋯*A*	*D*—H⋯*A*
C2—H2⋯N3^i^	0.93	2.66	3.468 (6)	146

Symmetry code: (i) 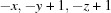.

## supplementary crystallographic information

### S1. Comments

Pyrimidines have inter­esting biological properties with applications as pesticides, pharmaceutical agents (Condon *et al.*, 1993; Maeno *et al.*, 1990) and are also inter­esting from a biochemical pint of view and applications of organoselenide compounds (Ip *et al.*, 1997). Pyrimidine derivatives have been developed as anti­viral agents, such as AZT, which is the anti-AIDS drug most widely used (Gilchrist, 1997). Recently, a new series of highly substituted pyrimidine herbicides have been reported (Selby *et al.*, 2002; Zhu *et al.*, 2007). In the present study, we were inter­ested in examining a derivative of pyrimidine with a methyl substituent surrounded by two chlorine atoms.

The molecular structure of the title compound is shown in Fig. 1. The molecule is planar, as is typical in benzenes substituted by halogen atoms and methyl groups, with an r.m.s. deviation for all non-H atoms of 0.009 Å. The largest deviation from the mean plane is 0.016 (4) Å for atom N3. The bond distances and bond angles in the molecule are similar to those reported for 5-methyl­pyrimidine (Furberg *et al.*, 1979).

In the crystal, molecules are linked by a pair of C—H···N hydrogen bonds forming inversion dimers (Table 1 and Fig. 2), enclosing an *R*^2^_2_(6) ring motif.

### S2. Synthesis and crystallization

The commercially available title compound (Sigma-Aldrich) was recrystallized from ethanol giving colourless prismatic crystals.

### S3. Refinement

Crystal data, data collection and structure refinement details are summarized in Table 2. A l l H atoms were localized in a difference Fourier map but introduced in calculated positions and treated as riding: C—H = 0.93–0.96 Å with *U*_iso_(H) = 1.5*U*_eq_(*C*-methyl) and 1.2*U*_eq_(C) for other H atoms.

### Crystal data

**Table:**

C_5_H_4_Cl_2_N_2_	*F*(000) = 328
*M**_r_* = 163.00	*D*_x_ = 1.575 Mg m^−^^3^
Monoclinic, *P*2_1_/*n*	Mo *K*α radiation, λ = 0.71073 Å
*a* = 7.463 (5) Å	Cell parameters from 776 reflections
*b* = 7.827 (5) Å	θ = 4.2–27.8°
*c* = 11.790 (5) Å	µ = 0.85 mm^−^^1^
β = 93.233 (5)°	*T* = 293 K
*V* = 687.6 (7) Å^3^	Prism, colourless
*Z* = 4	0.11 × 0.10 × 0.08 mm

### Data collection

**Table:**

Oxford Diffraction Xcalibur, Eos diffractometer	1228 independent reflections
Radiation source: Enhance (Mo) X-ray Source	791 reflections with *I* > 2σ(*I*)
Graphite monochromator	*R*_int_ = 0.099
CCD rotation images, thin slices ω scans	θ_max_ = 25.2°, θ_min_ = 3.1°
Absorption correction: multi-scan (*CrysAlis PRO*; Oxford Diffraction, 2013)	*h* = −8→8
*T*_min_ = 0.922, *T*_max_ = 0.934	*k* = −9→4
2347 measured reflections	*l* = −14→10

### Refinement

**Table:**

Refinement on *F*^2^	Primary atom site location: structure-invariant direct methods
Least-squares matrix: full	Secondary atom site location: difference Fourier map
*R*[*F*^2^ > 2σ(*F*^2^)] = 0.068	Hydrogen site location: inferred from neighbouring sites
*wR*(*F*^2^) = 0.173	H-atom parameters constrained
*S* = 1.01	*w* = 1/[σ^2^(*F*_o_^2^) + (0.0729*P*)^2^] where *P* = (*F*_o_^2^ + 2*F*_c_^2^)/3
1228 reflections	(Δ/σ)_max_ < 0.001
83 parameters	Δρ_max_ = 0.39 e Å^−^^3^
0 restraints	Δρ_min_ = −0.38 e Å^−^^3^

### Special details

**Table:**

Geometry. All e.s.d.'s (except the e.s.d. in the dihedral angle between two l.s. planes) are estimated using the full covariance matrix. The cell e.s.d.'s are taken into account individually in the estimation of e.s.d.'s in distances, angles and torsion angles; correlations between e.s.d.'s in cell parameters are only used when they are defined by crystal symmetry. An approximate (isotropic) treatment of cell e.s.d.'s is used for estimating e.s.d.'s involving l.s. planes.

### Fractional atomic coordinates and isotropic or equivalent isotropic displacement parameters (Å^2^)

**Table:**

	*x*	*y*	*z*	*U*_iso_*/*U*_eq_	
Cl14	0.47393 (16)	0.21816 (17)	0.57181 (8)	0.0686 (5)	
Cl16	0.45631 (17)	0.4430 (2)	0.13763 (9)	0.0853 (6)	
N1	0.2066 (5)	0.4832 (6)	0.2775 (3)	0.0604 (11)	
N3	0.2153 (5)	0.3882 (5)	0.4695 (3)	0.0579 (10)	
C2	0.1383 (6)	0.4657 (7)	0.3784 (4)	0.0661 (14)	
H2	0.0251	0.5123	0.3865	0.079*	
C4	0.3744 (5)	0.3225 (5)	0.4544 (3)	0.0458 (10)	
C5	0.4659 (5)	0.3309 (6)	0.3546 (3)	0.0458 (10)	
C6	0.3650 (6)	0.4175 (6)	0.2685 (3)	0.0514 (11)	
C51	0.6464 (6)	0.2553 (7)	0.3426 (4)	0.0666 (14)	
H51A	0.7041	0.3121	0.2823	0.100*	
H51B	0.6344	0.1359	0.3252	0.100*	
H51C	0.7175	0.2692	0.4124	0.100*	

### Atomic displacement parameters (Å^2^)

**Table:**

	*U*^11^	*U*^22^	*U*^33^	*U*^12^	*U*^13^	*U*^23^
Cl14	0.0846 (9)	0.0601 (9)	0.0604 (6)	−0.0009 (7)	−0.0019 (6)	0.0089 (5)
Cl16	0.0934 (11)	0.1053 (14)	0.0592 (7)	−0.0109 (9)	0.0219 (6)	0.0116 (6)
N1	0.057 (2)	0.061 (3)	0.063 (2)	0.005 (2)	0.0033 (16)	0.0012 (19)
N3	0.057 (2)	0.057 (3)	0.0602 (19)	0.000 (2)	0.0160 (15)	−0.0040 (17)
C2	0.050 (2)	0.074 (4)	0.075 (3)	0.008 (3)	0.005 (2)	−0.008 (3)
C4	0.045 (2)	0.039 (3)	0.0537 (19)	−0.003 (2)	0.0041 (16)	−0.0034 (18)
C5	0.042 (2)	0.039 (3)	0.057 (2)	−0.005 (2)	0.0089 (16)	−0.0056 (17)
C6	0.054 (2)	0.053 (3)	0.0475 (18)	−0.004 (2)	0.0070 (16)	−0.0023 (18)
C51	0.051 (3)	0.070 (4)	0.081 (3)	0.007 (3)	0.016 (2)	−0.002 (2)

### Geometric parameters (Å, º)

**Table:**

Cl14—C4	1.737 (4)	C4—C5	1.395 (5)
Cl16—C6	1.733 (4)	C5—C6	1.403 (6)
N1—C6	1.299 (6)	C5—C51	1.486 (6)
N1—C2	1.327 (5)	C51—H51A	0.9600
N3—C4	1.315 (5)	C51—H51B	0.9600
N3—C2	1.335 (5)	C51—H51C	0.9600
C2—H2	0.9300		
			
C6—N1—C2	115.4 (3)	C6—C5—C51	125.2 (4)
C4—N3—C2	114.8 (3)	N1—C6—C5	125.9 (3)
N1—C2—N3	126.8 (4)	N1—C6—Cl16	115.6 (3)
N1—C2—H2	116.6	C5—C6—Cl16	118.5 (3)
N3—C2—H2	116.6	C5—C51—H51A	109.5
N3—C4—C5	125.7 (4)	C5—C51—H51B	109.5
N3—C4—Cl14	115.1 (3)	H51A—C51—H51B	109.5
C5—C4—Cl14	119.2 (3)	C5—C51—H51C	109.5
C4—C5—C6	111.4 (4)	H51A—C51—H51C	109.5
C4—C5—C51	123.4 (4)	H51B—C51—H51C	109.5
			
C6—N1—C2—N3	−0.2 (8)	Cl14—C4—C5—C51	−0.2 (6)
C4—N3—C2—N1	−0.7 (8)	C2—N1—C6—C5	0.5 (8)
C2—N3—C4—C5	1.3 (7)	C2—N1—C6—Cl16	−179.0 (4)
C2—N3—C4—Cl14	−178.9 (4)	C4—C5—C6—N1	0.1 (7)
N3—C4—C5—C6	−1.1 (6)	C51—C5—C6—N1	179.5 (5)
Cl14—C4—C5—C6	179.2 (3)	C4—C5—C6—Cl16	179.6 (3)
N3—C4—C5—C51	179.5 (4)	C51—C5—C6—Cl16	−1.0 (7)

### Hydrogen-bond geometry (Å, º)

**Table:**

*D*—H···*A*	*D*—H	H···*A*	*D*···*A*	*D*—H···*A*
C2—H2···N3^i^	0.93	2.66	3.468 (6)	146

Symmetry code: (i) −*x*, −*y*+1, −*z*+1.

## References

[bb1] Altomare, A., Burla, M. C., Camalli, M., Cascarano, G. L., Giacovazzo, C., Guagliardi, A., Moliterni, A. G. G., Polidori, G. & Spagna, R. (1999). *J. Appl. Cryst.* **32**, 115–119.

[bb2] Condon, M. E., Brady, T. E., Feist, D., Malefyt, T., Marc, P., Quakenbush, L. S., Rodaway, S. J., Shaner, D. L. & Tecle, B. (1993). *Brighton Crop Prot. Conf. Weeds*, pp. 41–46 Alton, Hampshire, England: BCPC Publications.

[bb3] Furberg, S., Grøgaard, J. & Smedsrud, B. (1979). *Acta Chem. Scand.* **33b**, 715–724.

[bb4] Gilchrist, T. L. (1997). *Heterocycl. Chem.* 3rd ed., pp. 261–276. Singapore: Addison Wesley Longman.

[bb5] Ip, C., Lisk, D. J., Ganther, H. & Thompson, H. J. (1997). *Anticancer Res.* **17**, 3195–3199. 9413148

[bb6] Macrae, C. F., Bruno, I. J., Chisholm, J. A., Edgington, P. R., McCabe, P., Pidcock, E., Rodriguez-Monge, L., Taylor, R., van de Streek, J. & Wood, P. A. (2008). *J. Appl. Cryst.* **41**, 466–470.

[bb7] Maeno, S., Miura, I., Masuda, K. & Nagata, T. (1990). *Brighton Crop Protection Conference on Pests and Diseases*, pp. 415–422 Alton, Hampshire, England: BCPC Publications.

[bb8] Oxford Diffraction (2013). *CrysAlis PRO*. Oxford Diffraction Ltd., Abingdon, UK.

[bb9] Selby, T. P., Drumm, J. E., Coats, R. A., Coppo, F. T., Gee, S. K., Hay, J. V., Pasteris, R. J. & Stevenson, T. M. (2002). *ACS Symposium Series*, Vol. 800, Synthesis and Chemistry of Agrochemicals VI, pp. 74–84. Washington DC: American Chemical Society.

[bb10] Sheldrick, G. M. (2015). *Acta Cryst.* C**71**, 3–8.

[bb11] Spek, A. L. (2009). *Acta Cryst.* D**65**, 148–155. 10.1107/S090744490804362X PMC2631630 19171970

[bb12] Zhu, Y.-Q., Zou, X.-M., Li, G.-C., Yao, C.-S. & Yang, H.-Z. (2007). *Chin. J. Org. Chem.* **27**, 753–757.

