# Supplementary figures and images for: Crystal structure of 4,6-di­chloro-5-methyl­pyrimidine
Source: Acta Crystallogr E Crystallogr Commun. 2015 Dec 19;71(Pt 12):o1073–4. doi: 10.1107/S2056989015024020 (PMC4719985; doi:10.1107/S2056989015024020)

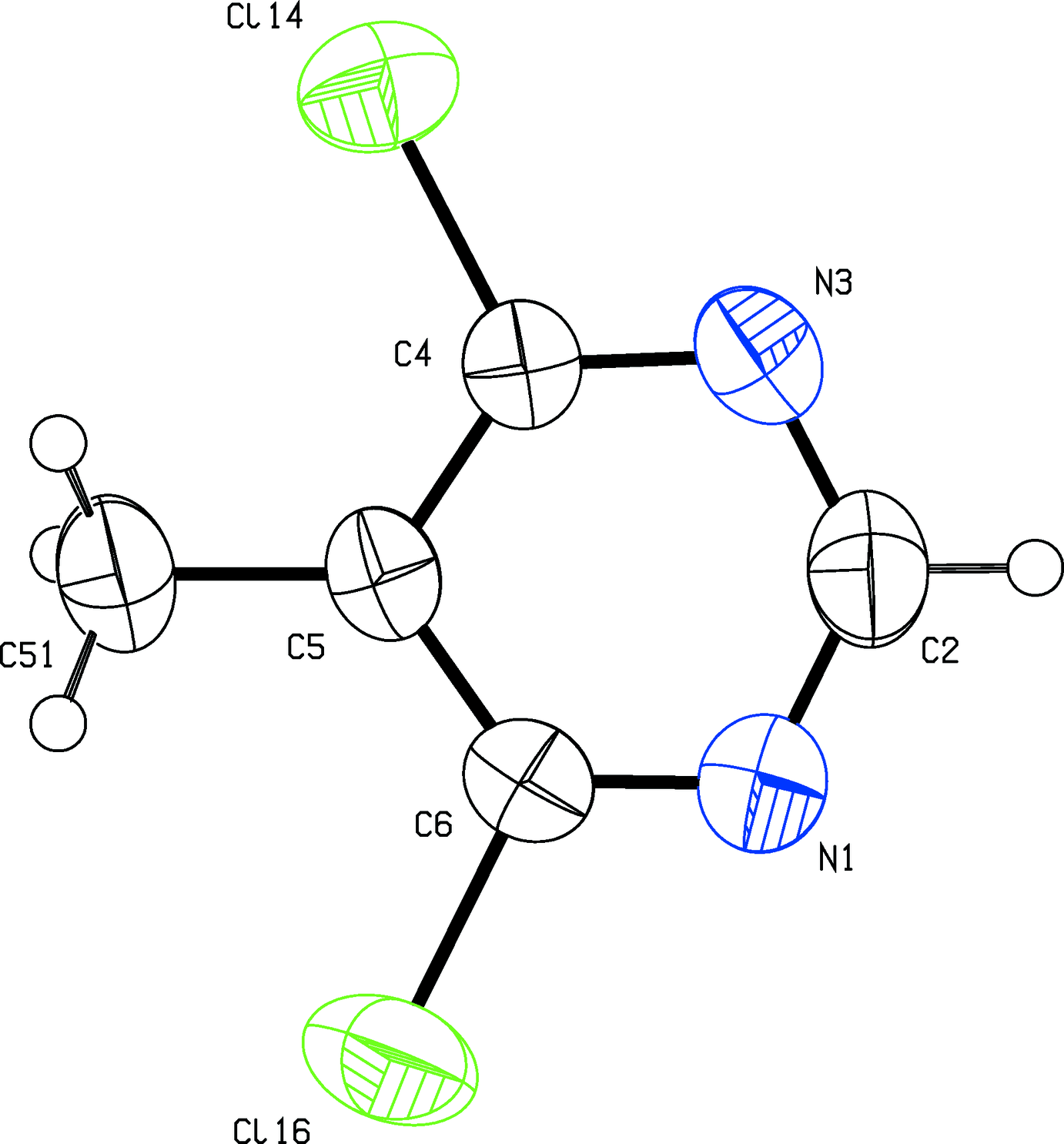

Supplement: Supplementary file 3 [file e-71-o1073-fig1.tif]

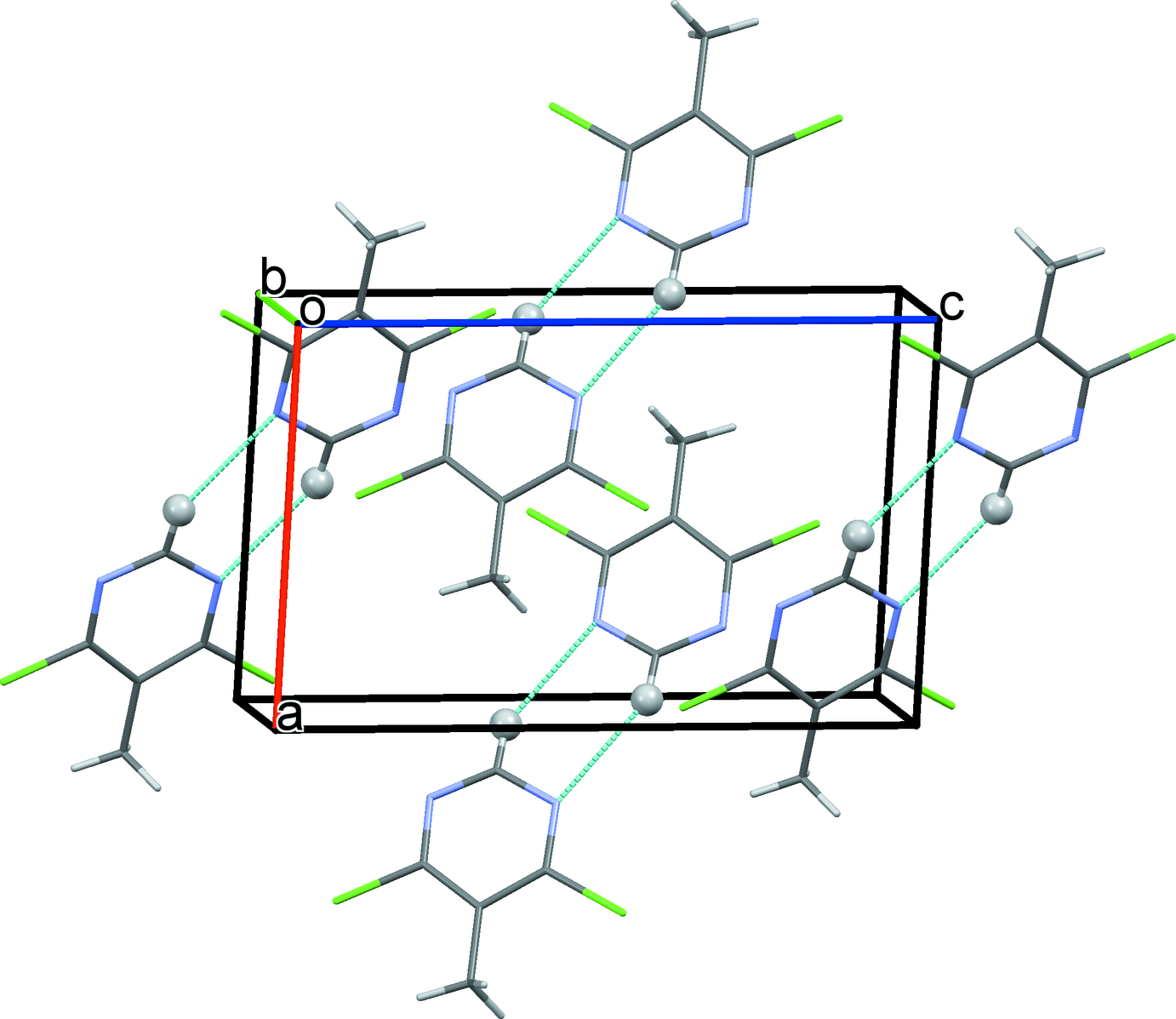

Supplement: Supplementary file 4 [file e-71-o1073-fig2.tif]
